# Anaemia in pregnancy and associated factors: a cross sectional study of antenatal attendants at the Sunyani Municipal Hospital, Ghana

**DOI:** 10.1186/s13104-017-2742-2

**Published:** 2017-08-11

**Authors:** Peter Anlaakuu, Francis Anto

**Affiliations:** 1 School of Public Health, University of Ghana, Legon, Ghana; 2 Sunyani Municipal Hospital, Sunyani, Ghana

**Keywords:** Anaemia in pregnancy, Malaria infection, Intake of fish and snail, Antenatal visits

## Abstract

**Background:**

Anaemia in pregnancy is an important health issue resulting in high maternal morbidity and mortality. The purpose of the current study was to identify factors associated with anaemia among pregnant women receiving antenatal care at the Sunyani Municipal Hospital in Ghana.

**Methods:**

A cross-sectional study involving pregnant women seeking antenatal care at the Sunyani Municipal Hospital was conducted between May and June, 2015. It involved the collection of data on socio demographic and obstetric variables, medical interventions and malaria infection, consumption of iron containing foods and supplements using a case record form and a structured questionnaire. Also, data on haemoglobin concentrations at first and current antenatal visit were collected. Bivariate and multivariate statistical analysis were done to determine factors associated with anaemia.

**Results:**

Out of the 316 participants, 129 (40.8%) were found to be anaemic (Hb <11.0 g/dl) at the time of their first ANC visit (mean Hb: 11.21 g/dl, range 6.8–15.1 g/dl). Seventy-nine (61.2%) of them had mild anemia (Hb 9.0–10.9 g/dl), 48 (37.2%) had moderate anemia (Hb 7.0–8.9 g/dl) whilst 2 (1.6%) had severe anemia (Hb <7.0 g/dl). During their most recent ANC visit, the prevalence of anaemia was found to be similar to that of the first visit with 131 (41.5%) of them being anaemic [mean Hb: 11.24 g/dl, range 8.10–14.5 g/dl]. The haemoglobin levels however improved significantly during the most recent visit compared to the first with none of the women being severely anaemic (Hb <7.0 g/dl). The prevalence of moderate anaemia reduced from 37.2% (CI 28.9–46.2) during the first visit to 19.1% (12.7–26.9) during the most recent visit, a reduction of 48.7%. Malaria infection, frequency at which one consumed fish/snails and gestational age at first ANC visit were the main factors found to be associated with anaemia among the pregnant women.

**Conclusions:**

Malaria infection, fish/snails intake and gestational age at first ANC visit were significantly associated with anaemia. Addressing these factors can reduce the incidence of anaemia in pregnancy.

**Electronic supplementary material:**

The online version of this article (doi:10.1186/s13104-017-2742-2) contains supplementary material, which is available to authorized users.

## Background

Anaemia is a major public health problem with about two billion people being anaemia worldwide [1]. The global prevalence of anaemia in pregnancy is estimated to be approximately 41.8% varying from a low of 5.7% in the United State of America to a high of 75% in Gambia [2]. Some women are anaemic even before they become pregnant and others become progressively anaemic during pregnancy [3]. Infectious diseases such as malaria, helminths infestations, and HIV have been implicated in the high prevalence of anemia in sub-Saharan Africa [4].

Anaemia is an important risk factor in pregnancy and it is associated with an increased incidence of both maternal and foetal morbidity and mortality. More than three percent of maternal mortality in Africa are directly attributable to anaemia [5]. Maternal anaemia also contributes to an increase in perinatal mortality, low birth weight, still birth and foetal wastage. Anaemia in pregnancy reduces tolerance to blood loss and leads to impaired function and cardiac failure [6].

Anemia prevalence data remains an important indicator in public health since anemia is related to morbidity and mortality in the population groups usually considered to be the most vulnerable; pregnant women and children under five. Anemia prevalence study is also useful to monitor the progress of reproductive health. Despite efforts being made to reduce the burden of anemia, its prevalence is still high in developing countries. Thus, the objective of this study was to determine factors associated with anemia among pregnant women who sought antenatal care (ANC) in the Sunyani Municipal Hospital, a facility that serves both rural and urban populations in the Brong Ahafo region of Ghana.

## Methods

### Study area

The study was conducted in the Sunyani Municipal Hospital in the Brong Ahafo region of Ghana. The hospital’s bed capacity is 63, with an average outpatient attendance of 1300 per day. There are 265 professional nurses, five doctors, six physician assistants, twenty-two midwives and eight community health nurses. Routine antenatal services provided include Intermittent Preventive Treatment for malaria in Pregnancy (IPTp), health education, immunization, and monitoring of haemoglobin levels of the women.

### Study design

A hospital based cross sectional study was conducted at the Sunyani Municipal Hospital from May to June 2015. All pregnant women aged 15 years and above who visited the antenatal clinic of the hospital during the period were eligible to participate in the study. Primary data were collected from the women as well as a review of their antenatal records.

### Sample size estimation and sampling

The sample size was estimated using the prevalence of anaemia in pregnancy reported for the region (29.0%) [7]. Using the Cochran formula, n = (Z^2^pq)/d^2^ [8], where: n = sample size, Z = the z-score that corresponds with 95% confidence interval (1.96), P = proportion of anaemia in pregnancy (29.0%, =0.29), q = proportion of antenatal attendants who are not anaemic(1–0.29%, =0.71), d = margin of error set at 5% (0.05), a sample size of n = 316 was estimated.

Thirteen participants were randomly selected on each day of data collection from an average of 60 antenatal attendants using a sampling interval of five. The first participant was randomly selected among the first five who reported for antenatal care on each day. The subsequent fifth was selected until 13 participants were enrolled for the day. Participants who did not consent to participate in the study were replaced with the next person following her. This was repeated until the required sample was obtained.

### Data collection methods and tools

Data on socio-demographic characteristics such as age, educational level, number of children, occupation, marital status, frequency of taking iron containing foods and bed net usage were collected directly from the mothers onto a questionnaire designed specifically for this study.

The questionnaire was developed based on the key indicators of the study and reviewed by colleagues with expertise in epidemiology, nutrition and malarialogy. Some variables that have been identified in earlier studies in Ghana and elsewhere in Africa to influence anaemia in pregnancy were included [3, 4]. The questionnaire consisted of partially categorised questions divided into three sections, namely: socio-demographic; nutritional and malaria prevention practices and previous and current obstetric history. The data were collected after the women had received ANC services for the day. The data collection was carried out by trained research assistants with university degrees, who are fluent in the local language Akan and English. For the purpose of accuracy, some relevant data were extracted from the ANC booklets. These included, number of ANC visits, gravidity, parity, haemoglobin concentration at first and current visits, gestational age at first and current ANC visit and administration of anti-helminths during current pregnancy. Additional data included malaria infection during current pregnancy, administration of iron supplementation and gestational age at which first (Intermittent Preventive Treatment in pregnancy (IPTp) was administered.

### Quality control

Quality control was conducted by pre-testing the questionnaire to determine its appropriateness and suitability for the study. This resulted in corrections, rephrasing of questions and rearrangement of sections in the questionnaire. Pre-testing was done using 20 ANC attendants over a period of 2 days (10/day) at the SDA hospital, a health facility also located in the Sunyani Municipality and providing similar health services. To ensure uniformity of the process, the two data collectors involved in the study were trained for 5 days on how to explain the study objectives, conduct the interviews and obtain informed consent. Data extracted from the ANC books were verified by a supervisor at the facility.

### Data processing and analysis

Data were entered into Epi Data version 3.1 and exported to Stata version 12 for analysis. Categorical variables were summarized into frequencies and proportions. Continuous variables were summarized into means and ranges. Continuous variables such as age were categorized into age groups, Hb values were categorized into anaemia (Hb <11.0 g/dl), mild anaemia (Hb 10–10.9 g/dl), moderate anaemia (7–9.9 g/dl) and severe anaemia (Hb <7 g/dl) [1]. Bivariate analysis was done using Pearson Chi square tests to assess significant differences between anaemia and categorical variables. Binary logistic regression was used to assess for factors associated with anaemia. Factors with p < 0.05 at 95% CI were considered statistically significant and therefore included in the multiple logistic regression model.

### Inclusion/exclusion criteria

All pregnant women aged 15 years and above with at least two antenatal visits and record of Hb concentration at first and current visit were eligible to participate in the study. Pregnant women who had history of blood transfusion (within the previous 2 weeks) and those who declined consent were excluded.

## Results

### Socio-demographic and obstetric characteristics of study participants

A total of 316 pregnant women aged 15–45 years (mean 28.42 years and SD ± 5.6 years) accessing antenatal care (ANC) services at the Sunyani Municipal Hospital participated in the study. One hundred and thirteen (35.7%) of them were aged 25–29 years, 163 (51.6%) had basic level education and 69.0% (218/316) were self-employed. Most of the women (57.9%, 183/316) were married (Table 1). As at the time of the study, 76.4% (120/316) of the women in their third trimester of pregnancy had made four or more ANC visits, whilst majority of those in their first trimester (83.3%, 15/316) were coming for their second visit. Ninety-four (29.7%) of the women had not delivered before, whilst 137 (43.4%) of them have had more than one delivery (Table 1), with 31.3% (99) of them carrying their third pregnancy.

**Table 1:** Background characteristics of study participants

Variable	Frequency (N = 316)	Percentage	
Age category (years)			
15–19	21	6.7	
20–24	61	19.3	
25–29	113	35.7	
30–34	78	24.7	
35 and above	43	13.6	
Highest educational level			
No formal education	22	7.0	
Primary	163	51.6	
Secondary	83	26.3	
Tertiary	48	15.2	
Occupation of participants			
Government worker	44	13.9	
Self employed	218	69.0	
Unemployed	30	9.5	
Other (students)	24	7.6	
Marital status			
Single	50	15.8	
Married	183	57.9	
Cohabitation	79	25.0	
Divorced/separation/widowed	4	1.3	
Parity			
Para zero	94	29.7	
Primigravidae	85	26.9	
Multigravidae	137	43.4	

No. of ANC visits	Gestational age		
	1st trimester	2nd trimester	3rd trimester
	n (%)	n (%)	n (%)
Two	15 (83.3)	51 (36.2)	12 (7.6)
Three	3 (16.7)	61 (43.2)	25 (15.9)
Four or more	0 (0)	29 (20.6)	120 (76.4)
Total	18 (100)	141 (100)	157 (100)

### Malaria prevention and food practices among the pregnant women

A total of 278 (88.0%) of the women indicated that they own at least one insecticide treated bed net, with 69.1% (192/278) of them sleeping under a net the night before the data collection. All the women had received Intermittent Preventive Treatment in Pregnancy involving sulfadoxine–pyrimethamine (IPTp-SP) during the most recent pregnancy with 80.1% (253/316) of them taking the first dose during the second trimester, whilst 63 (19.9%) of them received the drug during the third trimester. Most of the women (96.5%, 305/316), also received iron supplements. Out of the 316 women, only 76 (24.1%) had received anti-helminths medication during the current pregnancy. A total of 62 (19.6%) of the women have had malaria infection during the current pregnancy with most of them (66.1%) being infected during the second trimester (Table 2). Twenty (6.3%) of the women never ate eggs during their current pregnancy whilst 19 (6.0%) never consumed fish or snails during the current pregnancy.

**Table 2:** Medical interventions and malaria infections during pregnancy

Factors	Frequency N = 316	% [95% CI]
Bed net ownership		
Own a bed net	278	88.0 [83.9–91.3]
Does not own a bed net	38	12.0 [8.7–16.1]
Bed nets use		
Slept under bed net the previous night	192	60.8 [55.1–66.2]
Did not sleep under bed net last night	124	39.2 [33.8–44.9]
Gestational age at which IPT1 was taken		
Second trimester	253	80.1 [75.2–84.3]
Third trimester	63	19.9 [15.7–24.8]
Use of anti-helminthes		
No dewormer received	240	75.9 [70.8–80.6]
At least one dose of dewormer received	76	24.1 [19.4–29.2]
Malaria infection during pregnancy		
Been infected	62	19.6 [15.4–24.4]
Not been infected	254	80.4 [75.6–84.6]
Gestational age malaria infection occurred		
First trimester	8	12.9 [5.7–23.9]
Second trimester	41	66.1 [53.0–77.7]
Third trimester	13	21.0 [11.7–31.2]

### Prevalence of anaemia among the pregnant women

**Fig. 1:**
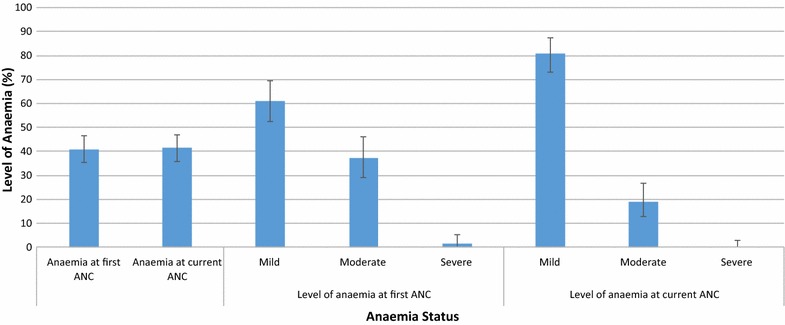
Anaemia among ANC attendants at the Sunyani Municipal Hospital, May 2015. The points plotted (*end of bars*) indicate the level of anaemia. The *vertical lines* show the corresponding 95% confidence intervals

Out of the 316 participants, 129 (40.8%) were found to be anaemic (Hb <11.0 g/dl) at the time of their first ANC visit (mean Hb: 11.21 g/dl, range 6.8–15.1 g/dl). Seventy-nine (61.2%) of them had mild anemia (Hb 9.0–10.9 g/dl), 48 (37.2%) had moderate anemia (Hb 7.0–8.9 g/dl) whilst 2 (1.6%) had severe anemia (Hb <7.0 g/dl). During their most recent ANC visit, the prevalence of anaemia was found to be similar to that of the first visit with 131 (41.5%) of them being anaemic (Hb <11.0 g/dl) [mean Hb: 11.24 g/dl, range 8.10–14.5 g/dl]. The prevalence of moderate anaemia reduced form 37.2% (CI 28.9–46.2) during the first visit to 19.1% (12.7–26.9) during the most recent visit, a reduction of 48.7% (Fig. 1).

### Factors associated with anaemia among the pregnant women

Three main factors were found to be associated with anaemia among the pregnant women. These were, malaria infection, frequency at which one consumed fish/snails [sources of protein] and gestational age at first ANC visit. The number of deliveries, gestational age at which IPTp-SP was taken and iron supplementation did not seem to be associated with anaemia in pregnancy in the current study.

There was a significant difference in the prevalence of anaemia between women who had malaria infection and those who did not, with those infected being 7.2 times more likely to be anaemic than those who were not infected (P = 0.008) (Tables 3, 4). Similarly, not eating fish/snails made the women more vulnerable to developing anaemia with those who did not take any fish/snails being 8.6 times more likely to become anaemic; with those who ate fish/snails for three or more times per week being more protected against anaemia (P = 0.02) (Tables 3, 4).

**Table 3:** Some factors contributing to anemia in pregnancy among ANC attendants in the Sunyani Municipal Hospital

Factors	Anaemia N (%)	Not anaemic N (%)	χ^2^	P
Number of ANC visits				
Two visit	37 (47.4)	41 (52.6)	5.31	0.07
Three visit	28 (31.6)	61 (68.4)		
Four or more visit	66 (44.3)	83 (55.7)		
Parity				
Para zero	45 (47.9)	49 (52.1)	2.943	0.230
Primigravidae	30 (35.3)	55 (64.7)		
Multigravidae	56 (40.9)	81 (59.1)		
Malaria infection during pregnancy				
Been infected	35 (56.5)	27 (43.5)	7.15	0.008
Not been infected	96 (37.8)	158 (62.2)		
Frequency of consuming fish/snail				
Never	12 (63.2)	7 (36.8)	8.55	0.04
Once a week	15 (50.0)	15 (50.0)		
Twice per week	52 (45.2)	63 (54.8)		
Three or more per week	52 (34.2)	100 (65.8)		
Gestational age at first visit				
First trimester	72 (36.2)	127 (63.8)	8.222	0.016
Second trimester	57 (49.6)	58 (50.4)		
Third trimester	2 (100)	0 (0)

**Table 4:** Association between anemia, dietary habit, ANC visits, iron supplementation, malaria prevention and infection

Variables	COR	95% CL	P	AOR	95% CI	P
IPTp-SP						
No SP taken	1.0					
At least one dose of SP	0.64	0.38–.07	0.09			
Frequency of taking meat, fish/snail						
Never	1.0			1.0		
Once a week	0.58	0.18–1.89	0.37	1.03	0.42–2.54	0.95
Twice per week	0.48	0.18–1.31	0.15	0.70	0.29–1.69	0.43
Three or more times per week	0.30	0.11–0.82	0.02	2.15	0.58–7.98	0.25
Iron supplementation taken during pregnancy						
Received iron supplementation	1.0					
Received no iron supplementation	0.8	0.23–2.79	0.73			
Gestational age at which IPT was taken						
Third trimester	1.0					
First trimester	0.76	0.43–1.31	0.32			
Second trimester	1.43	0.73–2.80	0.29			
Malaria infection during pregnancy						
Been infected	1.0			1.0		
Not been infected	0.47	0.27–0.82	0.008	0.47	0.25–0.90	0.021
Number of ANC visit						
Two visits	1.0			1.0		
Three visits	0.04	0.27–0.96	0.04	0.65	0.32–1.32	0.24
Four or more visits	0.51	0.51–1.53	0.65	0.89	0.48–1.61	0.70

There was also a significant difference in the level of anaemia between women who made their first ANC visit during the first trimester and those who made the first visit during the second or third trimesters. Women who visited during the first trimester were 8.2 times more protected than those who visited later during the pregnancy (P = 0.016) (Table 3).

## Discussion

Anemia in pregnancy is an important public health problem as it impacts not only on the pregnant woman but also significantly affects the unborn child. A cross sectional study was carried out in the Sunyani Municipal Hospital in Ghana to determine the prevalence of aneamia and associated factors among antenatal (ANC) attendants. The prevalence of anaemia in this study was 41.5%, with factors such as gestational age at first ANC visit, malaria infection and consumption of fish and snails being significantly association with the condition.

The level of anaemia found in this study was within global ranges (42–44%) [9] as well as ranges reported from other parts of Africa (40%) in Northwest Ethiopia [10], 57.3% in South Africa, 66% in Burkina Faso [11]. It is evident from the current study that the prevalence of anaemia in pregnancy is still high in the Sunyani Municipality despite various interventions including free distribution of insecticide treated nets [12], regular iron supplementation and improved antenatal care [13]. Although the level of anaemia in the current study is much lower than that reported by Dei-Adomakoh, and colleagues, it is high enough to be of public health concern as depending on the severity and duration of anemia and the stage of gestation, it can result in several adverse effects including low birth weight and preterm delivery [14].

Parasitic infections especially malaria and helminths during pregnancy have been associated with increased risk of maternal anaemia and adverse pregnancy outcomes [15]. It is well known that anaemia is a serious clinical manifestation of malaria and results from increased destruction of both infected and uninfected red blood cells due to membrane alterations [16] and also ingestion of the cytoplasm of the red blood cells by the *Plasmodium* parasite [17]. It was therefore not surprising that women who had malaria during pregnancy were about five times more likely to be anaemic than those who did not.

Several studies [18–22] have found association between malaria infection and anaemia in pregnancy. As infection by malaria parasites and destruction of red blood cells is central to the reproduction and survival of the parasite. This relationship between malaria infection and anaemia in pregnancy is critical in the health of women in their reproductive age especially in malaria endemic areas. The already existing interventions (ITN usage, IPTp-SP, iron supplementation, deworming, indoor residual spraying), would have to be strengthened as they have proven to be effective [22, 23] in earlier studies.

It is worth noting that most of the women made their first ANC visit during the first trimester of pregnancy with only a few (2%) making the first visit very late in the third trimester. Visiting ANC early in pregnancy reduced the likelihood of being anaemic. Early and regular antenatal visits are essential [24] as this could allow for the correction of anaemia that might exist even before the pregnancy. This essentially is achieved through iron supplementation as it is taken regularly throughout the pregnancy and supplies replenished during subsequent ANC visits. Thus, pregnant women who do not go for ANC regularly may not have the full benefit of iron supplementation. Also, early ANC visits will allow for prompt treatment of malaria infections which are usually more common during the early stages of pregnancy [25] and predisposes the pregnant woman to anaemia [26].

Pregnant women who consumed fish or snails regularly were less likely to become anaemic compared to those who never consumed fish or snails. Nutritional anaemia is known to be the most common type of anaemia [27] with pregnant women who take less than two meals a day, less diverse meals or less meat being more likely to be anaemic. Thus, balanced diet involving meat and vegetable [28] and eggs [29] is essential during pregnancy in preventing anaemia.

The prevalence of anaemia is known to vary with the seasons and highest at the end of the malaria transmission season (just before the start of dry season). The main limitation of this study is that it was carried out at the beginning of the high malaria transmission season and covered only 2 months. Similarly, since eligibility was based on having had at least two ANC visits including the most recent one, for some women both visits (i.e., first and most recent) could be in the same trimester. This could reduce the true change in prevalence of anaemia between the first visit to the most recent one. These limitations notwithstanding, the study has been able to identify some of the factors associated with anaemia in pregnancy in the study area (Additional files 1, 2).

## Conclusions

Three main factors, malaria infection, frequency at which one consumed fish/snails and gestational age at first ANC visit were found to be significantly associated with anaemia among the pregnant women. The number of deliveries, gestational age at which IPTp-SP was taken and iron supplementation did not seem to be associated with anaemia in pregnancy in the current study. Strengthening malaria prevention, improving upon dietary intake and regular monitoring of haemoglobin levels during pregnancy could help reduce anaemia in this vulnerable population. This is in line with the current policy of free long lasting insecticidal net distribution to pregnant women during ANC visits to prevent malaria and also the increase in the recommended minimum IPT-p doses—from two to three—by the WHO and three to five by the Ghana Health Service. Well designed health education messages and programmes could also help improve uptake and usage of these intervention. Through the existing comprehensive ANC services, individualized dietary programmes can be drawn for identified pregnant women all directed at reducing anaemia and improving pregnancy outcomes.

## Additional files

**Additional file 1.** Anaemia in pregnancy-Sunyani.

**Additional file 2.** Data collection form.

## Abbreviations

ANC: Antenatal Clinic
AOR: adjusted odds ratio
COR: crude odds ratio
CL: confidence interval
WHO: World Health Organization
Hb: haemoglobin
IPT: Intermittent Preventive Treatment
MCH: Maternal and Child Health
SP: sulphadoxine pyrimethanine
SMH: Sunyani Municipal Hospital
OPD: Outpatient Department
