# Supplementary material for: Anaemia in pregnancy and associated factors: a cross sectional study of antenatal attendants at the Sunyani Municipal Hospital, Ghana
Source: BMC Res Notes. 2017 Aug 11;10:402. doi: 10.1186/s13104-017-2742-2 (PMC5553653; doi:10.1186/s13104-017-2742-2)
Supplement: Supplementary file 2 — Additional file 2. Data collection form. [file 13104_2017_2742_MOESM2_ESM.docx]

**ADDITIONAL FILE 2**

**DATA COLLECTION FORM**

**FACTORS ASSOCIATED WITH ANAEMIA IN PREGNANT WOMEN ATTENDING ANTENATAL CLINIC IN SUNYANI MUNICIPAL HOSPITAL**

| **#** | **Basic data** | | | | | | | | | | | |
| --- | --- | --- | --- | --- | --- | --- | --- | --- | --- | --- | --- | --- |
| **1** | Form No. |  | | |  | | | |  | | | FORM_NO |
| **2** | Date of interview |  |  |  | |  |  |  | |  |  | DATE |
| **3** | Age of respondent (yrs) |  | | | | |  | | | | | AGE |
| **4** | Highest level of education | 1. No formal education  2. Basic  3. Secondary  4. Tertiary | | | | | | | | | | EDUC |
| **5** | Occupation | 1. Government worker  2. Self employed  3. Unemployed  77. Other | | | | | | | | | | OCCU |
| **6** | Marital status | 1. Single  2. Married  3. Divorced  4. Widowed | | | | | | | | | | MARRI |
| **7** | Community of residence | 1. Rural  2. Urban | | | | | | | | | | RESID |
| **8** | Ethnic group | 1. Akan  2. Mo  3. Gruma  4. Konkomba  5. Fulani  6. Gonja  77. Other (specify)……………………. | | | | | | | | | | ETHNIC |
|  | **Nutrition and malaria prevention** | | | | | | | | | | | |
| **9** | How many times in a week do you take an egg during this pregnancy? | 1. Never  2. Once  3. Twice  4. Three or more times | | | | | | | | | | EGG |

| 10 | How many times in a week do you take meat/fish/snails during this pregnancy? | 1. Never  2. Once  3. Twice  4. Three or more times | MEFISN |
| --- | --- | --- | --- |
| 11 | How many times in a week do you take green leafy vegetable/fruits during this pregnancy? | 1. Never  2. Once  3. Twice  4. Three or more times | VEG_FRU |
| 10 | Have you taken iron supplementation during this pregnancy? | 1. Yes  2. No | FE-SUPP |
| 11 | If yes how many times? | 1. Once  2. Sometimes  3. Always | FE_TIMES |
| 12 | Do you have an ITN? | 1. Yes  2. No | HAVE_NET |
| 13 | If yes, did you under it last night? | 1. Yes  2. No | SLEEP_NET |
| 14 | Have you had malaria during this pregnancy? | 1. Yes  2. No | MALARIA |
| 15 | If yes, at what time of the pregnancy? (weeks) | …………………… | MALA_TIME |
|  | **Obstetric and anaemia history** | | |
| 16 | At what time of the pregnancy did you come for the first ANC? (weeks) | …………………… | FIRST_ANC |
| 17 | What is the current gestational age? (weeks) | …………………… | CURRENT_ANC |
| 18 | How many times have you come for ANC during the current pregnancy? | …………………… | ANC_VISITS |
| 19 | Have you received any deworming medicine during the current pregnancy? | 1. Yes  2. No | DEWORM |
| 20 | Have you received IPTp during current pregnancy? | 1. Yes  2. No | RES-IPTP |

| 21 | At what time of the pregnancy did you receive the first dose of IPTp? (weeks) | …………………… | | | | FIRST_IPTP |
| --- | --- | --- | --- | --- | --- | --- |
| 22 | Gravidity (number of pregnancies) | …………………… | | | | GRAVIDA |
| 23 | Parity (number of children) | …………………… | | | | PARITY |
| 24 | What is the interval between the last child and the current pregnancy? (yrs) | ……………………  88. N/A | | | |  |
| 25 | Hb at first ANC |  |  | **.** |  | FIRST_HB |
| 26 | Hb at current ANC |  |  | **.** |  | CURR_HB |
